# Endocrine mucin-producing sweat gland carcinoma with regional metastases in an African American female

**DOI:** 10.1016/j.jdcr.2023.03.012

**Published:** 2023-04-05

**Authors:** Alexzandra Mattia, Anthony Thompson, William Harris Green, Armand B. Cognetta

**Affiliations:** a Florida State University College of Medicine, Tallahassee, Florida; b Dermatology Associates of Tallahassee, Tallahassee, Florida

**Keywords:** EMPSGC, endocrine mucin-producing sweat gland carcinoma, HCTZ, hydrochlorothiazide, metastasis, MMS, mohs micrographic surgery

*To the Editor:* We read with interest the successful experience of Meltzer et al^1^ on the treatment of endocrine mucin-producing sweat gland carcinoma (EMPSGC) with Mohs micrographic surgery. The case emphasized the importance of timely diagnosis, as the rate of metastasis for EMPSGC is often higher compared to other non-melanoma skin cancers. Akin to our experience, the lesion mimicked a chalazion in clinical appearance. We report a novel and aggressive case of EMPSGC in an African American female.

A female in her 60s presented to ophthalmology with a firm and painful lesion on her right upper eyelid over a 2-year period. Ophthalmology empirically treated the lesion as a chalazion with incision and drainage on 3 occasions. Pathology was not obtained. The procedures resulted in continued growth and septation, prompting referral to dermatology. Physical examination revealed a papillated, pebbly tumor on the right lateral canthus (Fig 1). No adenopathy or masses were noted of the head and neck. Shave biopsy of the lesion demonstrated dermal basophilic cellular nodule comprised of ducts and mucin. A diagnosis of EMPSGC was confirmed, and Mohs micrographic surgery was used to extirpate the tumor and achieve margin control. Plastic surgery was consulted for reconstruction.

**Fig 1:**
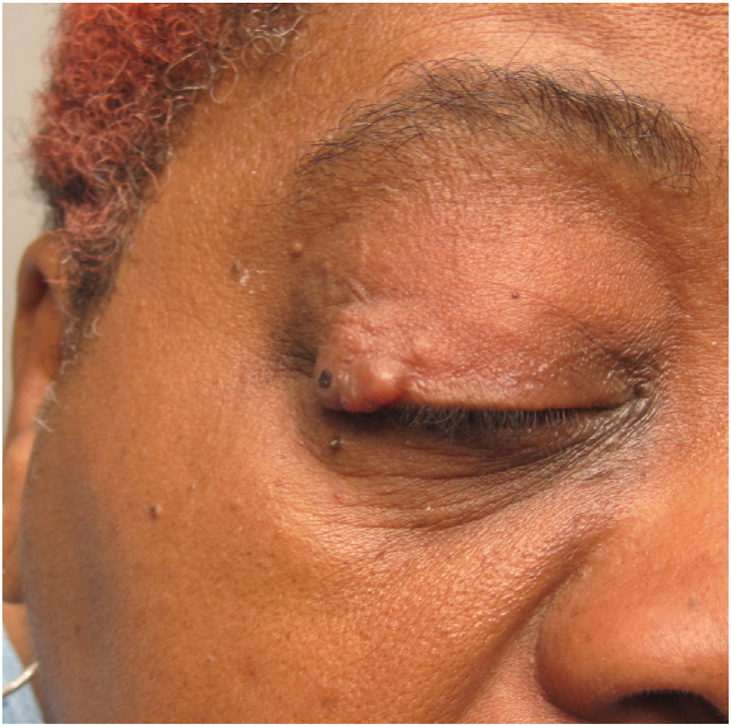
Physical examination of the eyelid revealed a papillated tumor measuring 15 mm × 15 mm in size with a 0.5 cm mucosal component extending from the mucosa and pushing on the inside of the eye.

Nine months later, our patient noticed a lump on her right cheek. A computed tomography scan revealed an enhancing, lobulated mass in the right parotid gland (Fig 2). No intracranial metastasis was present. Punch biopsy of the right preauricular lesion revealed a papillary growth pattern and large cribriform nests with extracellular mucin (Fig 3). Cells were diffusely positive for synaptophysin, Pan-Keratin, cytokeratin 7, epithelial membrane antigen, and carcinoembryonic antigen (Fig 4) and negative for chromogranin, neural cell adhesion molecule, and cytokeratin 20. Findings were consistent with metastatic EMPSGC, and the patient underwent right parotidectomy with adjuvant radiotherapy.

**Fig 2:**
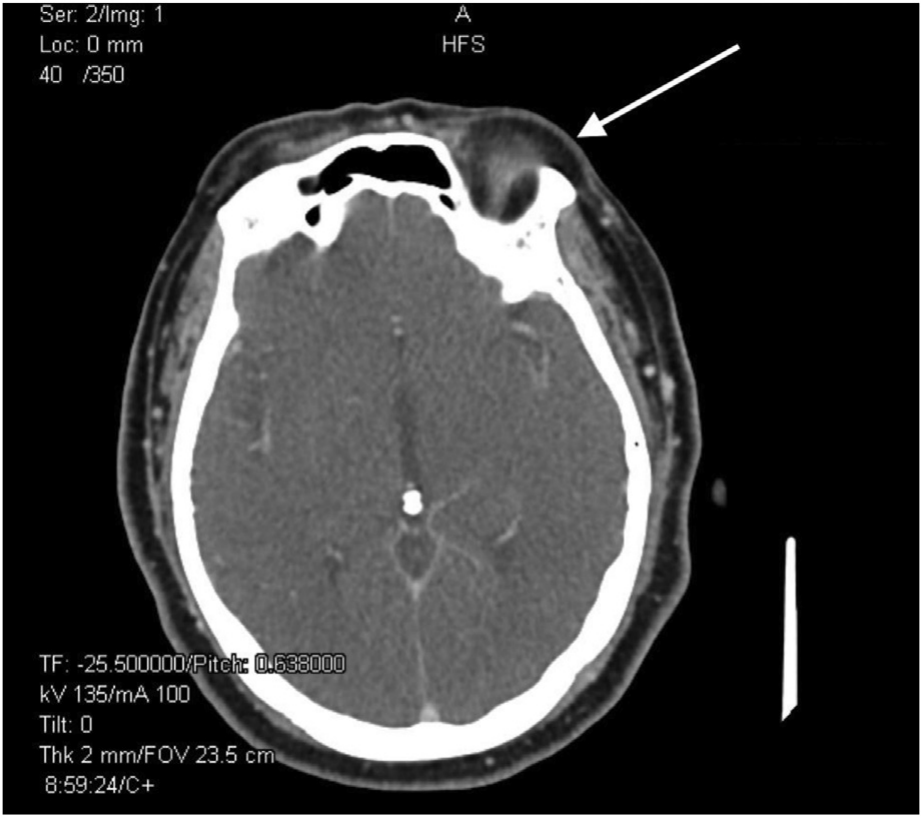
Axial image of computed tomography scan displayed a 2.6 cm transversely by 1.7 cm anteroposteriorly mass arising at the anterior aspect of the *right* parotid gland, extending to the subcutaneous region compatible with lymph node metastasis (*arrow* points to mass).

**Fig 3:**
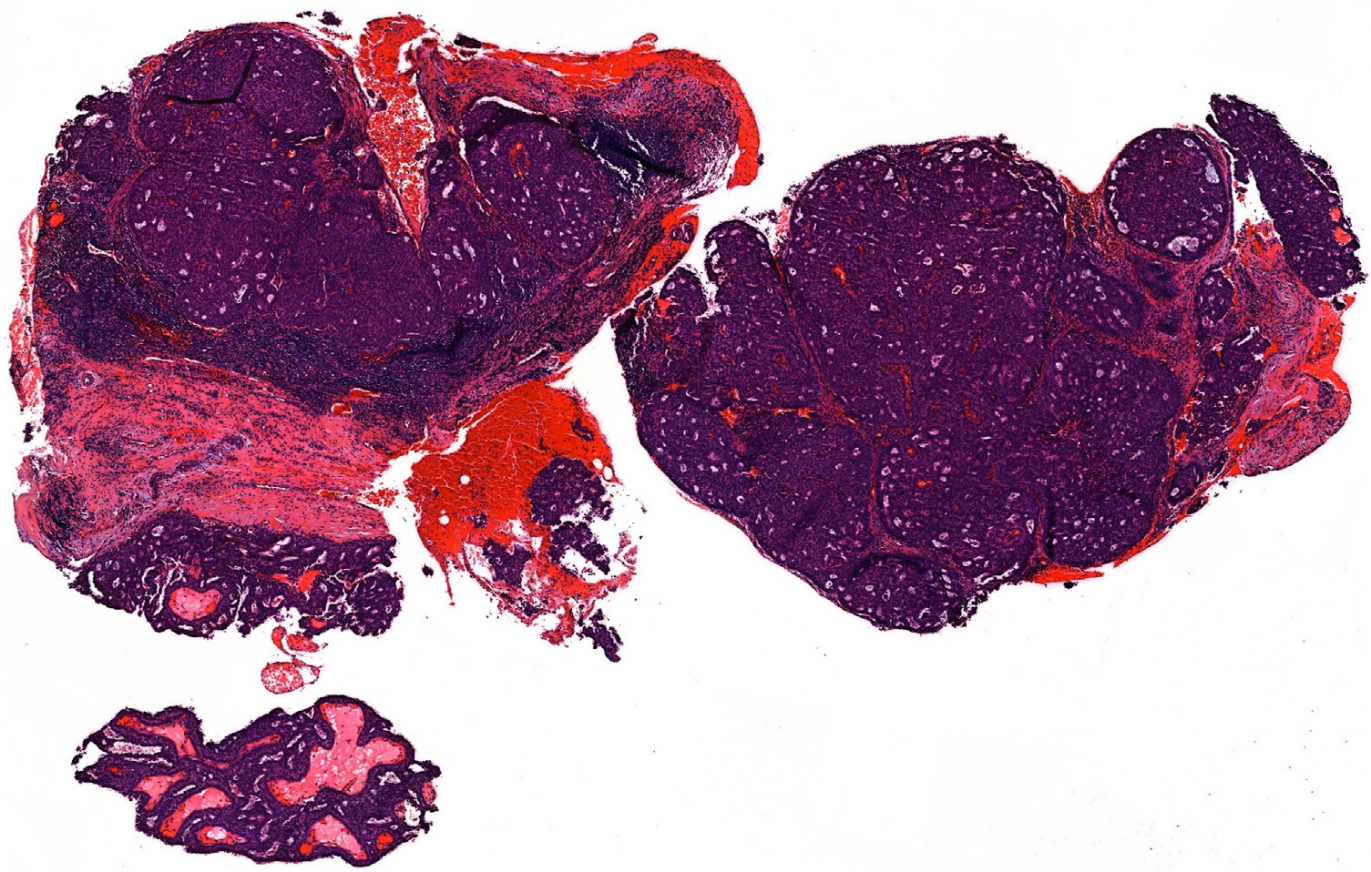
Histological examination of tumor cells displayed nests of atypical glandular cells with a papillary growth pattern (hematoxylin-eosin ×25).

**Fig 4:**
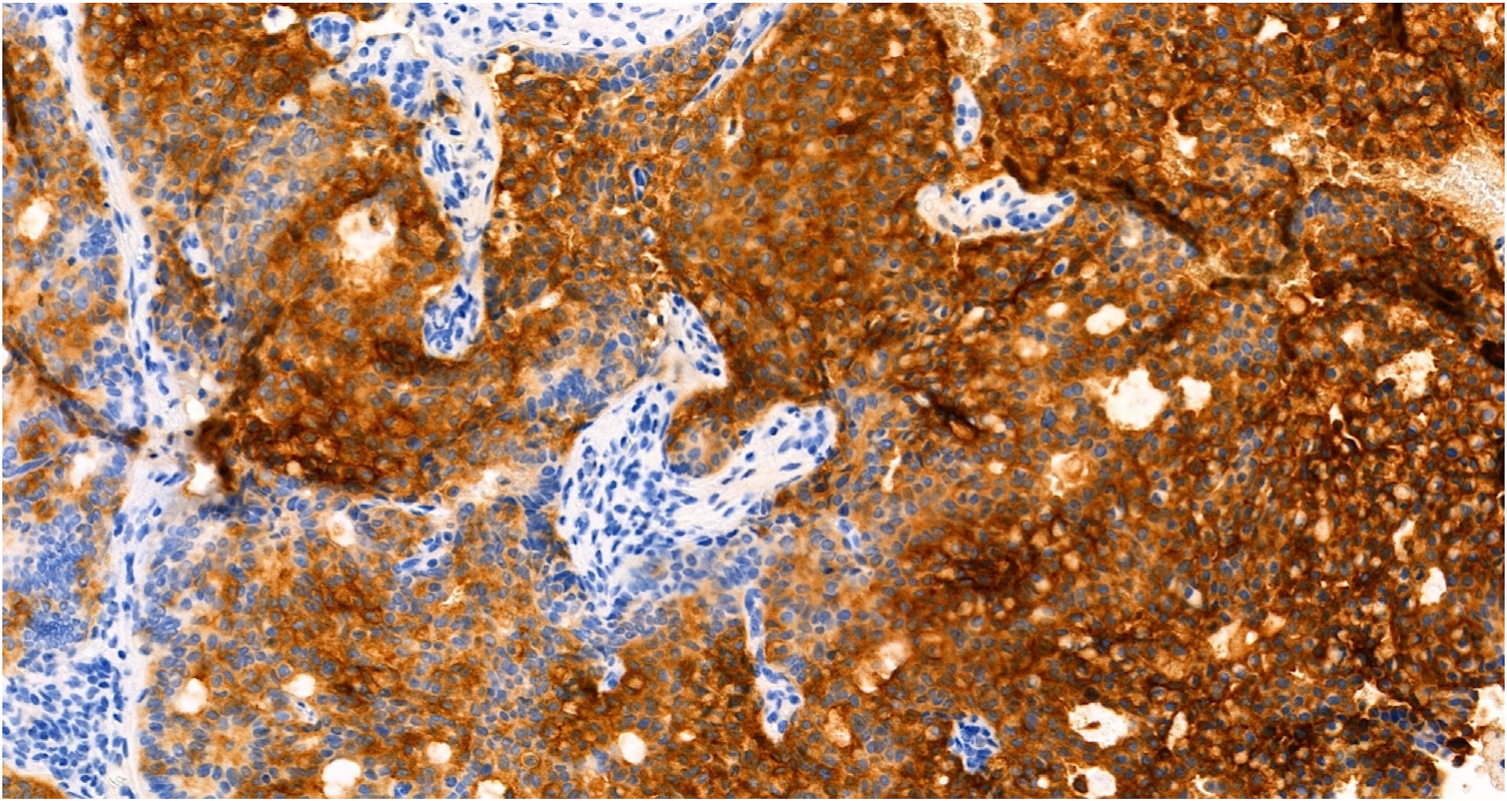
Metastatic parotid tumor cells stained diffusely positive for CEA (×20).

Six years after parotidectomy, ultrasound-guided fine needle aspiration revealed a 1.3 cm × 1.2 cm nodule in the right supraclavicular fossa with identical histology. Our patient underwent surgical extirpation of this second metastatic tumor and proton-beam radiation therapy.

EMPSGC is a cutaneous adnexal tumor, often presenting as an asymptomatic, slow-growing nodule or papule with a predilection for the eyelids in females.^1^ Two literature reviews published in February of 2013 and June 2021 documented a total of 20 cases and 190 cases, respectively, demonstrating an approximately 850% increase in the 8-year span.^2^^,^^3^ Three metastatic cases have been reported, all occurring at least 7 years from initial diagnosis.^3^

A possible correctable cause of our patient’s case included her 15-year history of photosensitizing hydrochlorothiazide. Cognetta et al^4^ demonstrated an increased risk of nonmelanoma skin cancer in a dose response fashion, especially with greater than 5 years of usage. Our patient discontinued hydrochlorothiazide upon consultation with her primary care physician at the time of initial presentation.

Our case corroborates previous reports suggesting this entity may be more aggressive than initially believed, introduces hydrochlorothiazide as a modifiable risk factor, and describes the second case of EMPSGC in an African American female.^5^ The nonspecific clinical findings may be a diagnostic challenge in patients of color, posing a threat for delayed diagnosis.

## Conflicts of interest

None disclosed.
